# Proteomic studies in *VWA1*‐related neuromyopathy allowed new pathophysiological insights and the definition of blood biomarkers

**DOI:** 10.1111/jcmm.18122

**Published:** 2024-04-23

**Authors:** Mohammed Athamneh, Nassam Daya, Andreas Hentschel, Andrea Gangfuss, Tobias Ruck, Adela Della Marina, Ulrike Schara‐Schmidt, Albert Sickmann, Anne‐Katrin Güttsches, Marcus Deschauer, Corinna Preusse, Matthias Vorgerd, Andreas Roos

**Affiliations:** ^1^ Department of Neurology, Heimer Institute for Muscle Research University Hospital Bergmannsheil, Ruhr‐University Bochum Bochum Germany; ^2^ Department of Clinical Science, Faculty of Medicine Yarmouk University Irbid Jordan; ^3^ Leibniz‐Institut für Analytische Wissenschaften‐ISAS‐e.V. Dortmund Germany; ^4^ Department of Pediatric Neurology, Centre for Neuromuscular Disorders, Centre for Translational Neuro‐ and Behavioral Sciences University Duisburg‐Essen Essen Germany; ^5^ Department of Neurology, Medical Faculty Heinrich Heine University Düsseldorf Düsseldorf Germany; ^6^ Department of Neurology Technical University of Munich, School of Medicine Munich Germany; ^7^ Institute of Neuropathology, Charité–Universitätsmedizin Berlin, corporate member of Freie Universität Berlin Humboldt Universität zu Berlin, Berlin Institute of Health Berlin Germany; ^8^ Children's Hospital of Eastern Ontario Research Institute University of Ottawa Ottawa Canada

**Keywords:** BET1, HNRNPDL, NEFM and PHGDH, neuromyopathy, Von Willebrand factor a domain containing 1 protein

## Abstract

Bi‐allelic variants in *VWA1*, encoding Von Willebrand Factor A domain containing 1 protein localized to the extracellular matrix (ECM), were linked to a neuromuscular disorder with manifestation in child‐ or adulthood. Clinical findings indicate a neuromyopathy presenting with muscle weakness. Given that pathophysiological processes are still incompletely understood, and biomarkers are still missing, we aimed to identify blood biomarkers of pathophysiological relevance: white blood cells (WBC) and plasma derived from six *VWA1*‐patients were investigated by proteomics. Four proteins, BET1, HNRNPDL, NEFM and PHGDH, known to be involved in neurological diseases and dysregulated in WBC were further validated by muscle‐immunostainings unravelling HNRNPDL as a protein showing differences between *VWA1*‐patients, healthy controls and patients suffering from neurogenic muscular atrophy and *BICD2*‐related neuromyopathy. Immunostaining studies of PHGDH indicate its involvement in apoptotic processes via co‐localisation with caspase‐3. NEFM showed an increase in cells within the ECM in biopsies of all patients studied. Plasma proteomics unravelled dysregulation of 15 proteins serving as biomarker candidates among which a profound proportion of increased ones (6/11) are mostly related to antioxidative processes and have even partially been described as blood biomarkers for other entities of neuromuscular disorders before. CRP elevated in plasma also showed an increase in the extracellular space of *VWA1*‐mutant muscle. Results of our combined studies for the first time describe pathophysiologically relevant biomarkers for *VWA1*‐related neuromyopathy and suggest that *VWA1*‐patient derived blood might hold the potential to study disease processes of clinical relevance, an important aspect for further preclinical studies.

## 1 INTRODUCTION

Neuromuscular disorders may vary considerably in their pathogenesis, clinical manifestations and disease course.^1^ A precise molecular diagnosis is often complicated by the genetic and phenotypic variation known to be present in a significant subset of neuromuscular disorders. On the other hand, the presence of similar symptoms and pathomorphological hallmarks among some of the different entities hints towards common pathomechanisms, an important aspect for the definition of therapeutic concepts targeting similar pathophysiological cascades.^2^ Whereas over the last decade developments in sequencing technologies and the establishment of worldwide collaborations have gradually elucidated the origins of various hereditary neuromuscular diseases such as neuropathies and myopathies, analytical approaches including protein profiling enabled to delineate pathways, biomarkers and therapeutic targets.^2^ Here, proteomics (liquid chromatography coupled to mass spectrometry) became a valuable technique to investigate thousands of proteins on the level of peptides (unique for each of the quantified proteins) by making use of a minimal amount of starting material. Based on the function of dysregulated proteins, significant information can be obtained regarding pathophysiologies taking place in the investigated biomaterial.

Molecular genetics and biochemical findings have progressively emphasized the extracellular matrix's significance in the aetiology of neuromuscular disorders.^3^, ^4^ The von Willebrand factor A‐domain‐related protein (WARP) encoded by the *VWA1* gene, is an orphan extracellular matrix (ECM) protein, expressed in a subset of ECM structures. Muscular tissues and peripheral nerves express WARP which is interacting with collagen VI and perlecan.^5^, ^6^ In a *Vwa1* knockout mouse model, Warp deficiency has been reported to impair proper structure and function of peripheral nerves.^7^ Of note, exome sequencing studies revealed different bi‐allelic loss‐of‐function variants in *VWA1* as a novel pathogenic cause of a neuromyopathic disorder with child‐ or adulthood onset of proximal and distal muscular weakness, predominantly of the lower limbs as a prominent clinical characteristic. This is accompanied by myopathological and neurophysiological findings indicative of combined neurogenic and myopathic pathology.^5^ In this context, Pagnamenta and colleagues estimated that bi‐allelic variants in *VWA1* may even be responsible for up to 1% of unexplained cases with clinical suspicion of hereditary motor neuropathy in Europeans.^7^ Gable and colleagues postulated that this presumed high frequency may be associated with a wide spectrum of disease features and severity and along this line reported on two cases from nonconsanguineous families presenting in early childhood with lower extremity weakness and prominent foot deformities, upper motor neuron signs and abnormal gait phenotypes based on bi‐allelic variants in *VWA1* thus expanding the clinical picture.^8^

On a general note, WARP‐function is currently still poorly understood^9^ and especially the knowledge of WARP regarding its impact in the aetiology of neuromuscular disorders is primarily based on limited data. Moreover, there is no common consent if the muscular affection is a secondary pathology based on vulnerability and dysfunction of motoric axons or if a primary muscle pathology is rather part of the clinical picture.^5^, ^7^ Our research aimed to further extend current knowledge of the underlying pathophysiology of *VWA1*‐related neuromyopathy by identifying proteins affected by the loss of functional WARP and along this line to identify suitable marker proteins. Hereby, we were directed at proteins that (i) may contribute to the manifestation of neurological symptoms and (ii) along this line provide further evidence for a primary muscle cell vulnerability as well as (iii) hold the potential to be investigable in a minimal invasive manner. Latter aspect is important for the monitoring of therapeutic interventions in the future. Functional and biochemical research of neurological illnesses is often limited by the availability of appropriate biomaterial. Notably, patient‐derived white blood cells have been shown to represent suitable in vitro models to study the nature of rare neurological disorders overcoming the scarcity of tissues vulnerable in these diseases.^10^, ^11^, ^12^ Thus, here in the context of the first biomarker study of *VWA1*‐related neuromyopathy, we investigated white blood cells to define marker proteins with pathophysiological relevance in skeletal muscle as a tissue clinically affected by the presence of pathogenic *VWA1*‐variants. For that purpose, proteomic profiling was carried out on white blood cells derived from six *VWA1*‐patients of two unrelated families. By adopting paradigmatic results of our proteomic discovery study to muscle biopsy specimen derived from one of these patients and a further unrelated *VWA1*‐case. Moreover, proteomic profiling on plasma samples derived from these six patients was carried out to unravel further blood biomarkers.

## 2 PATIENTS, MATERIALS & METHODS

### 2.1 *VWA1* ‐patients included in the study

Demographic, molecular genetic and clinical data of patients included in our study^5^ is presented in Table 1. The labelling of patients presented in this study is used according to Table 1 throughout the manuscript. This study was approved by the Local Ethics Committee of the University Hospital Essen (19‐9011‐BO). Patients have been examined by experienced neuropediatricians and neurologists.

**TABLE 1:** Demographic data of *VWA1*‐patients included in our study. For patient seven, only the muscle biopsy was included. These seven patients were already part of the gene discovery study.^5^

*VWA1* patients					
Family	Individual	Genetic defect	Age of onset (years)	Age at last visit (years)	Clinical findings at last visit
1	1	*VWA1*: c.62_71dup c.879del compound heterozygous	3	16	Weakness of foot dorsiflexion with difficulties of standing on heels and bilateral pes equinus
	2		2	20	Weakness of foot dorsiflexion with difficulties of standing on heels and bilateral congenital pes equinovarus
2	3	*VWA1*: c.252del homozygous	43	66	Weakness of foot dorsiflexion with difficulties of standing on heels, proximal and distal leg weakness and proximal arm muscle weakness
	4		54	55	Weakness of foot dorsiflexion with difficulties of standing on heels
	5		46	62	Weakness of foot dorsiflexion with difficulties of standing on heels
	6		41	48	Weakness of foot dorsiflexion with difficulties of standing on heels, proximal and distal leg weakness & proximal arm muscle weakness
3	7	*VWA1*: c.62_71dup homozygous	40	46	Weakness of foot dorsiflexion with difficulties of standing on heels and bilateral pes cavus

### 2.2 Purification of white blood cells and subsequent proteomic analysis

**FIGURE 1:**
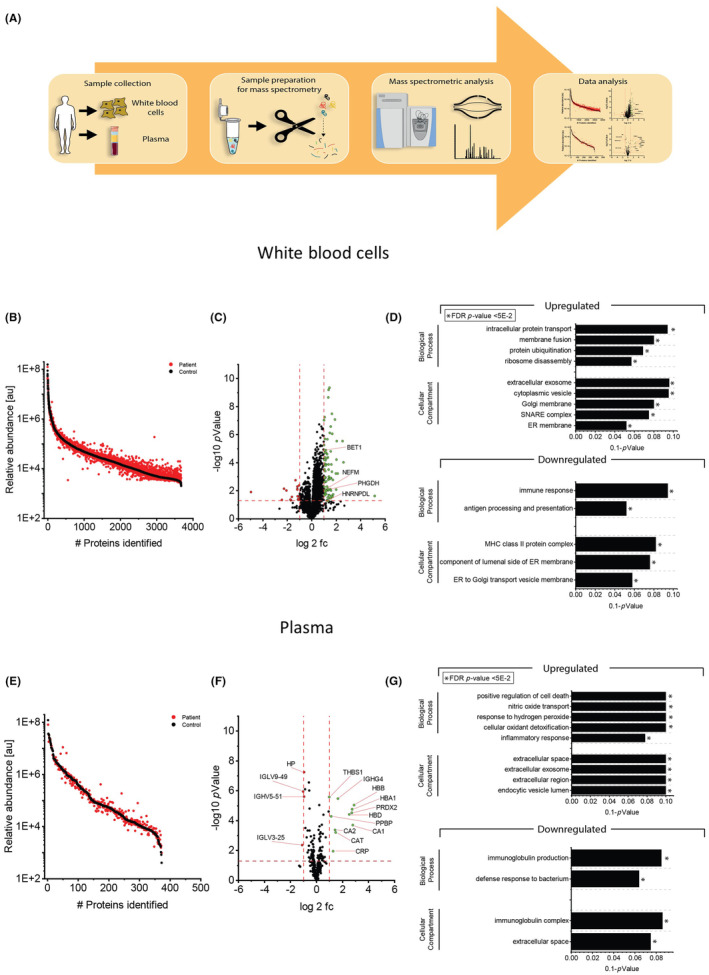
Proteomic studies on *VWA1*‐patient derived blood samples. (A) Results of GTex‐based in silico analysis of *VWA1* expression/transcript abundance in different tissues and cellular populations. (B) VWA1 protein abundances presented as ratios with respect to GAPDH level in in house generated spectral libraries (protein catalogues) of fibroblasts and skeletal muscle. (C) Schematic representation of the applied workflow to study the proteomic signatures of white blood cells and plasma from the same blood sample. (D) Abundance plot for proteomic profiling data obtained on white blood cells showing the dynamic range of all identified proteins. This is based on their relative quantification of the three highest abundant peptides for each protein, allowing protein comparison within an experiment. All identified proteins of the control (black) are sorted with decreasing abundance while the patient (red) was plotted in the same order to directly compare the different abundances. All identified proteins cover a dynamic range of eight orders of magnitude. (E) Volcano plot for proteomic findings obtained in white blood cells highlighting statistically significant increased proteins (green dots) as well as decreased proteins (red dots). Fc, fold change. Four proteins of particular neuromuscular relevance are highlighted. (F) Results of GO‐Term based in silico studies of proteomic findings in white blood cells show that increased proteins impact on intracellular protein transport, membrane fusion, protein ubiquitination and ribosome disassembly. Cellular compartments affected by increased protein abundances include membranes of the ER‐Golgi network as well as SNARE complex, extracellular exosomes and cytoplasmic vesicles (upper panel). Decreased proteins impact on immune response along with antigen processing and presentation and also affect the ER‐Golgi network (lower panel). (G) Abundance plot for proteomic profiling data obtained on plasma showing the dynamic range of all identified proteins. This is based on their relative quantification of the three highest abundant peptides for each protein, allowing protein comparison within an experiment. All identified proteins of the control (black) are sorted with decreasing abundance while the patient (red) was plotted in the same order to directly compare the different abundances. All identified proteins cover a dynamic range of eight orders of magnitude. (H) Volcano plot for proteomic findings obtained in plasma highlighting statistically significant increased proteins (green dots) as well as decreased proteins (red dots). (I) GO‐Term based in silico studies showed that increased proteins are indicative for positive regulation of cell death, oxidative stress burden (nitric oxide transport, response to hydrogen peroxide and cellular oxidant detoxification) and inflammatory response and impact on extracellular regions and endocytic vesicles as cellular compartments whereas decreased proteins are also indicative for immune response and affect the immunoglobulin complex and the extracellular space. Full names of proteins depicted in this figure are listed in Table 2 and Table S1.

White blood cells isolated and purified from 7.5 mL freshly collected EDTA‐blood^11^ derived from six *VWA1*‐patients: two siblings carrying the compound heterozygous c.62‐71dup and c.879del variants (family 1; see Table 1) and four relatives carrying the homozygous c.252del variant (family 2; see Table 1) in addition to a total of nine gender‐ and age‐matched controls (Figure 1C). None of the control individuals presented with any sign of disease at the time of blood sampling. Purified cells were snap‐frozen in liquid nitrogen and stored at −80°C until further processing for proteomic profiling.

### 2.3 Sample preparation for proteomic profiling

#### 2.3.1 White blood cell samples

Two hundred microlitres of lysis buffer containing 50 mM TEAB (pH 8.5), 5% SDS and cOmplete ULTRA protease inhibitor (Roche) were added to the snap frozen samples. To ensure complete lysis a sonication step using an ultrasonic probe (30 s, 1 s/1 s, amplitude 40%) followed by centrifugation at 4°C and 20,000 ***g*** for 15 min was conducted. The protein concentration of the supernatant was determined for each sample by BCA assay according to the manufacturer's protocol. Disulfide bonds were reduced by addition of 10 mM TCEP at 37°C for 30 min, and free sulfhydryl bonds were alkylated with 15 mM IAA at room temperature (RT) in the dark for 30 min. 100 μg protein of each sample was then used for proteolysis using the S‐Trap mini protocol (Protifi) using a protein to trypsin ratio of 20:1. The incubation time for trypsin was 2 h at 47°C. Proteolysis was stopped using formic acid to acidify the sample (pH <3.0). After the included clean‐up procedure, samples were dried using a vacuum concentrator. Each sample was dissolved in 0.1% TFA adjusting the peptide concentration to 0.5 μg/μL.

#### 2.3.2 Plasma samples

For plasma samples 10 μL of each sample was transferred into a new reaction tube. Lysis and protein denaturation was achieved by adding the lysis buffer mentioned above for preparation of white blood cells. No BCA was conducted as we calculate with a protein concentration of 70 μg/μL for each plasma sample. Reduction of disulfide bonds, alkylation and protein digestion was carried out as described in 1.1 by using the S‐TRAP mini protocol. The samples were dried and dissolved the same way as described above for processing of white blood cells.

#### 2.3.3 Quality control

After desalting, all proteolytic digests were checked for complete digestion using a monolithic column separation (PepSwift monolithic PS‐DVB PL‐CAP200‐PM, Dionex) on an inert Ultimate 3000 HPLC (Dionex, Germering, Germany) by direct injection of 0.5 μg sample. For this, a binary gradient (solvent A: 0.1% TFA, solvent B: 0.08% TFA, 84% ACN) of 5%–12% B in 5 min and then of 12%–50% B in 15 min was used at a flow rate of 2.2 μL/min and 60°C. The UV traces were recorded at 214 nm.^13^

### 2.4 Mass spectrometry

#### 2.4.1 Analysis of white blood cell samples

All samples were measured in data independent mode (DIA). The analysis was performed with an Ultimate 3000 nano RSLC system coupled to an Orbitrap Fusion Lumos mass spectrometer (all Thermo Scientific). For each measurement, 1 μg of peptides from each sample was pre‐concentrated on a 100 μm × 2 cm C18 trapping column for 10 min using 0.1% TFA (v/v) at a flow rate of 20 μL/min, followed by separation on a 75 μm × 50 cm C18 main column (both Pepmap, Thermo Scientific) with a 120‐min LC gradient of 3%–35% B (84% ACN in 0.1% FA) at a flow rate of 250 nL/min. An appropriate amount of iRT standard peptides (Biognosys) was added to each sample before starting the measurement. MS survey scans were acquired from 300 to 1100 m/z at a resolution of 60,000 FWHM, followed by MS/MS using 24 DIA windows, each covering a range of 25 m/z (with 1 m/z overlap) at a resolution of 30,000. The polysiloxane ion at 445.12 m/z was used as a lock mass. The CID spectra were recorded with a normalized collision energy of 32% and an activation time of 10 ms.

#### 2.4.2 Analysis of plasma samples

The analysis of all plasma samples was carried out by using an UltiMate 3000 RSLC nano UHPLC coupled to a QExactive HF mass spectrometer. The total amount of peptide applied was always 1 μg. Each sample was first transferred to a 75 μm × 2 cm, 100 Å, C18 pre column with a flow rate of 10 μL/min for 20 min. followed by a separation on the 75 μm × 50 cm, 100 Å, C18 main column with a flow rate of 250 nL/min and a linear gradient consisting of solution A (99.9% water, 0.1% formic acid) and solution B (84% acetonitrile, 15.9% water, 0.1% formic acid) where the pure gradient length was 120 min (3%–45% solution B). The gradient was applied as follows: 3% B for 20 min, 3%–35% for 120 min, followed by three washing steps each ranging to 95% buffer B for 3 min. After the last washing step, the instrument was allowed to equilibrate for 20 min. The acquisition of MS data was performed in DIA (data independent acquisition) mode using an in house build spectral library. Each sample analysed was mixed with an appropriate amount of iRT standard (Biognosys). Full MS scans were acquired from 300 to 1100 m/z at a resolution of 60,000 (Orbitrap) using the polysiloxane ion at 445.12002 m/z as lock mass. The automatic gain control (AGC) was set to 3E6 and the maximum injection time to 20 ms. Full MS scans were followed by 23 DIA windows, each covering a range of 28 m/z with 1 m/z overlap, starting at 400 m/z, acquired at a resolution 30,000 (Orbitrap) with an AGC set to 3E6 and nCE of 27 (CID).

### 2.5 Proteomic data analyses

#### 2.5.1 Protein identification

For analysis of samples acquired by nano‐LC‐MS/MS in DIA mode, data were submitted to Spectronaut software (Biognosys) and analysed using a library‐based search. A spectral library created in‐house was used as library. Search and extraction settings were kept as default (BGS Factory settings). Human proteome data from UniProt (www.uniprot.org) with 20,374 entries were selected as the proteome background.

For reliable label‐free quantification, only proteins with ≥2 unique peptides were considered for further analysis. Subsequently, average normalized abundances (determined using Spectronaut) were calculated for each protein and used to determine the ratio between patient samples and corresponding controls. Finally, Student's *t*‐test *p*‐values were calculated for each protein using MS Excel.

### 2.6 GO‐term and STRING analysis

To get a detailed overview of the biological processes, a GO‐Term analysis was performed. The online software DAVID (Database for Annotation, Visualization and Integrated Discovery) was used for this analysis.^14^, ^15^ Only significantly, regulated proteins (*p* ≤ 0.05) with either positive or negative regulation (fold change of 2 or higher and fold change of 0.5 or lower) were included in the analysis. GO‐Terms were checked for biological process, molecular function and cellular component and the results were manually filtered for relevant results. STRING network analyses (https://string‐db.org/) were performed to decipher a functional interplay of proteins dysregulated in blood cells and plasma.

### 2.7 Immunostaining studies on muscle biopsies derived from *VWA1* ‐ and disease control patients

To validate proteomic findings obtained on white blood cells, particularly with regard to dysregulated proteins of neurological relevance, immunofluorescence studies were carried out on 7 μm cryosections of muscle biopsy specimens derived from two *VWA1*‐patients and two controls as described previously.^16^ For this purpose, the following antibodies were utilized: anti‐BET1 (1:100; rabbit; Invitrogen, PA5‐88961), anti‐HNRNPDL (1:100; rabbit; Biozol USB‐482905), anti‐NEFM (1:100; mouse; Abcam [NF‐09] (ab7794)) and anti‐PHGDH (1:100; rabbit; Genetex, GTX101948) as well as anti‐CASP3 (1:100; mouse; Abcam, ab2171), anti‐neonatal Myosin (1:100; rabbit; Novocastra/Leica, NCL‐MHCn) and anti‐p62 (1:100; mouse; Abcam, ab56416). Spectrin staining utilising the anti‐SPEC antibody (1:100; mouse; Nococastra/Leica, NCL‐SPEC1) was carried out to visualize the sarcolemma. For immunofluorescence staining the following secondary antibodies were used: Alexa Fluor 488 Goat Anti‐Mouse IgG (Jackson ImmunoResearch; #115‐545‐146), Alexa Fluor Goat Anti‐Rabbit IgG (Jackson ImmunoResearch; #111‐545‐144), Alexa Fluor 594 Goat Anti‐Mouse IgG (Jackson ImmunoResearch; #115‐585‐068), Cy3‐conjugated Goat Anti‐Rabbit IgG (Jackson ImmunoResearch; #111‐165‐003) and DAPI (Thermo Scientific; #62248).

Immunohistochemistry staining of CD20, MUM1, CD138 and CRP was carried out as follows: skeletal muscle tissue of *VWA1*‐patients and controls were adapted to room temperature and blocked with goat serum for 30 min at room temperature. The primary antibody was applied for 2 h at room temperature. After washing in PBS for 2 × 5 min the secondary antibody goat anti‐mouse was added for 1 h at RT, dilution 1:100. After the final washing step, visualisation was reached with DAB (diaminobenzidine), followed by fixation with alcohol and xylol. For the staining procedure the following dilutions were utilized for the respective antibodies: anti‐CD20 (1:200; mouse; DAKO M0755), anti‐MUM1 (1:50; mouse; DAKO M7259), anti‐CD138 (1:30; mouse; DAKO M7228) and anti‐CRP (1:100; mouse; Proteintech 66250‐Ig). For this procedure a peroxidase detection kit (DCS; #PD000RP) containing a mixture of secondary antibodies was used.

## 3 RESULTS

### 3.1 Study of WARP‐abundances in different cellular populations/ tissues

To address the abundance of WARP (encoded by *VWA1*) in different cellular populations and complex tissues, we first carried out GTex‐based in silico studies (https://gtexportal.org; based on transcript level) revealing highest expression in the central nervous system and lowest in skeletal muscle and blood (Figure 1A). As this result is not in perfect accordance with the cellular vulnerability (neuromyopathy) upon presence of pathogenic *VWA1*‐variants, we screened our in‐house generated spectral libraries for skeletal muscle and fibroblasts for VWA1/WARP‐abundances in comparison to the level of GAPDH in terms of ratios and could confirm its expression with approximately two‐fold of magnitude lower abundance that GAPDH (Figure 1B).

### 3.2 Proteomic signature of *VWA1* ‐mutant white blood cells

Proteomic profiling allows the quantification of thousands of proteins along with the identification of a variety of protein dysregulations in a single experiment and thus holds the potential to unravel pathophysiological processes in an unbiased manner.^17^ In this study, global proteomics on whole protein extracts of white blood cells derived from six *VWA1*‐cases in addition to a total of nine gender‐ and age‐matched controls was applied (patients 1 to 6 in Table 1; Figure 1C). Based on a data‐independent approach, we quantified 2381 proteins with a p‐ANOVA below 0.05 (Figure 1D) A total of 101 proteins (4.2% of the statistically significant quantified proteins) showed a dysregulation: 16 proteins were decreased (5 quantified based on at least two unique peptides) whereas 85 were increased (16 quantified based on at least two unique peptides) (Figure 1E). A list of these proteins is presented in Table S1. The proteomic data have been deposited to the ProteomeXchange Consortium via the PRIDE^18^ partner repository with the dataset identifier PXD040226. Regarding the increased proteins, four (among others), BET1, HNRNPDL, NEFM and PHGDH (Figure S1A), have frequently been linked to the aetiology of diseases along the neuromuscular axis and were thus selected for further validation studies. Of note, these proteins are functionally interconnected to other dysregulated proteins as illustrated in our STRING network analysis focusing on all proteins dysregulated in *VWA1*‐patient derived cells. A GO‐term based in silico analysis aimed to delineate pathophysiological processes upon loss of functional *VWA1* expression. Increased proteins are indicative for altered intracellular protein transport and membrane fusion as well as increased protein ubiquitination and ribosome disassembly (Figure 1F). Cellular compartments affected by increased protein abundances include membranes of the ER‐Golgi network as well as the SNARE complex, extracellular exosomes and cytoplasmic vesicles (Figure 1F, upper panel). Decreased proteins are indicative for an altered immune response along with antigen processing and presentation (Figure 1F, lower panel). Here, decreased proteins also affect the ER‐Golgi network (Figure 1F).

### 3.3 Proteomic signature of plasma derived from *VWA1* ‐patients

Proteomic profiling of plasma samples derived from six *VWA1*‐patients (Figure 1C) allowed the robust quantification of 266 proteins (Figure 1G) and revealed the statistically significant dysregulation of 15 proteins. Hereby, 11 proteins were increased and 4 decreased compared to the signature of control samples (Figure 1H). A list of these proteins is presented in Table 2. A GO‐term based in silico analysis aimed to delineate pathophysiological processes mirrored in plasma samples derived from *VWA1*‐patients. Increased proteins are indicative for positive regulation of cell death, oxidative stress burden (nitric oxide transport, response to hydrogen peroxide and cellular oxidant detoxification) and inflammatory response and impact on extracellular regions and endocytic vesicles as cellular compartments (Figure 1I). Decreased proteins are also indicative for immune response and affect the immunoglobulin complex and the extracellular matrix (Figure 1I).

**TABLE 2:** List of proteins significantly dysregulated in *VWA1*‐patient derived plasma. Protein functions were extracted from UniProt (www.uniprot.org).

UniProt accession #	Protein name	Number of unique peptides identified	Ratio patient/control	*p*‐value	Function
Increased proteins					
P68871	Haemoglobin subunit beta (HBB)	7	7.41	0.000009	Functions as an endogenous inhibitor of enkephalin‐degrading enzymes
P00915	Carbonic anhydrase 1 (CA1)	8	6.94	0.000197	Catalyses the reversible hydration of carbon dioxide
P69905	Haemoglobin subunit alpha (HBA)	8	6.70	0.000017	Involved in oxygen transport from the lung to the various peripheral tissues
P32119	Peroxiredoxin‐2 (PRDX2)	5	6.59	0.000029	Plays a role in cell protection against oxidative stress by detoxifying peroxides and as sensor of hydrogen peroxide‐mediated signalling events
P02042	Haemoglobin subunit delta (HBD)	4	5.82	0.000038	Involved in oxygen transport from the lung to the various peripheral tissues
P01861	Immunoglobulin heavy constant gamma 4 (IGHG4)	6	3.14	0.000003	Constant region of immunoglobulin heavy chains
P04040	Catalase (CATA)	5	2,80	0,000628	Protects cells from the toxic effects of hydrogen peroxide and promotes growth of cells including T‐cells and B‐cells
P00918	Carbonic anhydrase 2 (CA2)	2	2,73	0.000423	Catalyses the reversible hydration of carbon dioxide
P02741	C‐reactive protein (CRP)	4	2,46	0.011229	Promotes phagocytosis and complement fixation through its calcium‐dependent binding to phosphorylcholine
P02775	Platelet basic protein (CXCL7)	5	2,20	0.000051	Stimulates mitosis, glycolysis, intracellular cAMP accumulation, prostaglandin E2 secretion, and synthesis of hyaluronic acid and sulfated glycosaminoglycan
P07996	Thrombospondin‐1 (TSP1)	15	2,01	0.000002	Mediates cell‐to‐cell and cell‐to‐matrix interactions
Decreased proteins					
P00738	Haptoglobin (HPT)	11	0.52	0.000000	Captures, and combines with free plasma haemoglobin to allow hepatic recycling of heme iron
A0A0C4DH38	Immunoglobulin heavy variable 5–51 (HV551)	2	0.52	0.000002	V region of the variable domain of immunoglobulin light chains that participates in the antigen recognition
A0A0B4J1Y8	Immunoglobulin lambda variable 9–49 (LV949)	2	0.49	0.000001	V region of the variable domain of immunoglobulin light chains that participates in the antigen recognition
P01717	Immunoglobulin lambda variable 3–25 (LV325)	2	0.47	0.004457	V region of the variable domain of immunoglobulin light chains that participates in the antigen recognition

### 3.4 Abundance and distribution of blood biomarker proteins in *VWA1* ‐mutant muscle

**FIGURE 2:**
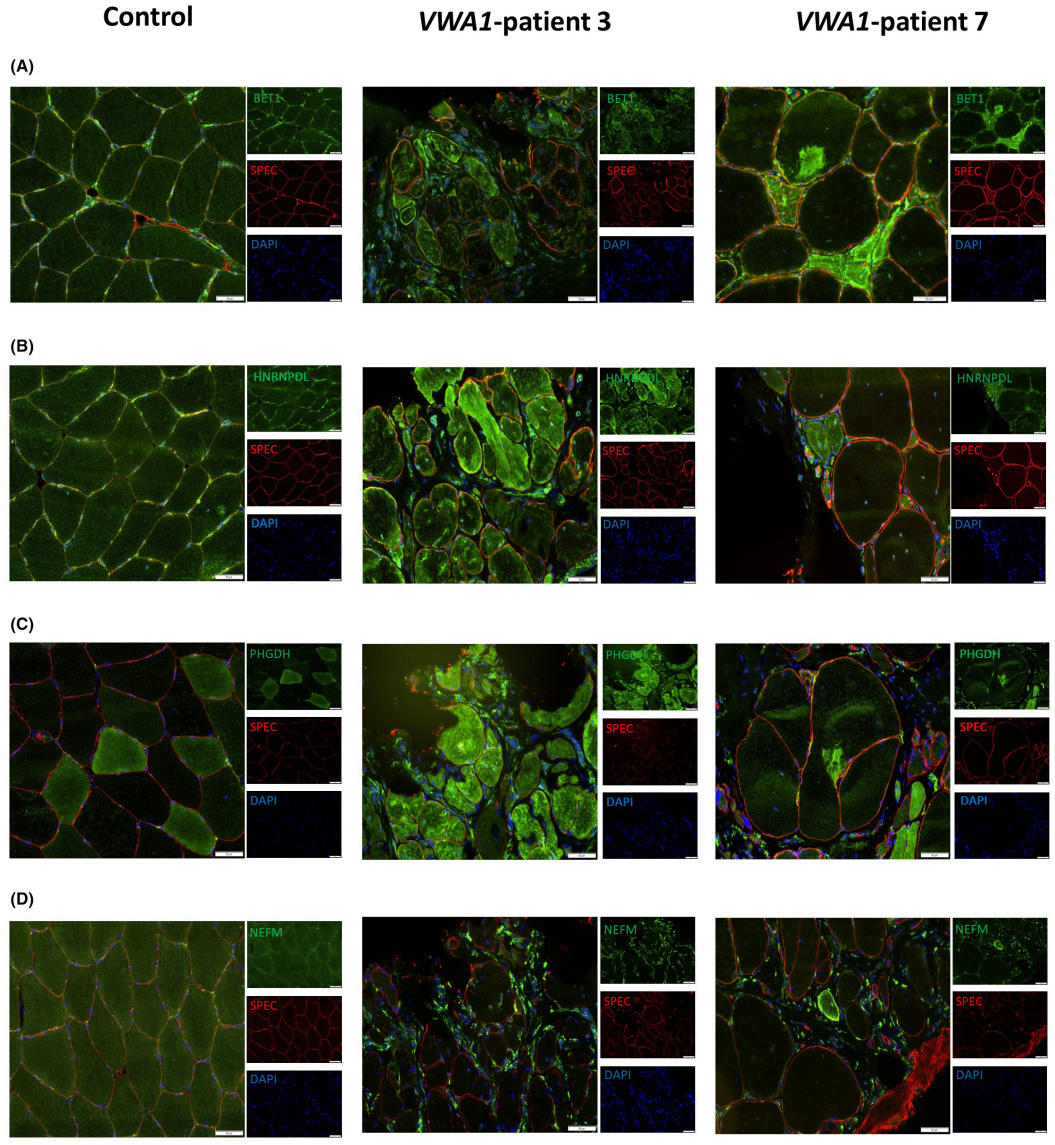
Immunofluorescence findings in *VWA1*‐patient derived muscle biopsies. (A) Immunofluorescence studies of BET1 (green) showed a sarcoplasmic increase accompanied by the presence of focal dot‐like structures in patient‐derived muscle cells compared to muscle cells derived from a control case. Spectin (SPEC) staining (red) visualizes the sarcolemma. (B) Immunostaining of HNRNPDL also revealed a sarcoplasmic increase accompanied by the presence of dot‐like immunoreactive structures in patient‐derived muscle cells compared to control cells. Spectin (SPEC) staining (red) visualizes the sarcolemma. (C) *VWA1*‐mutant muscle cells show a generalized sarcoplasmic increase with the presence of focal accumulations of PHGDH (green) in comparison to non‐mutant muscle cells. Spectin (SPEC) staining (red) visualizes the sarcolemma. (D) Immunofluorescence‐based studies of NEFM revealed a sarcoplasmic increase only in few myofibres in addition to a considerable increase in extra muscular cells localized within the extracellular matrix in *VWA1*‐mutant muscle compared to controls. One representative control biopsy is shown for the different staining studies. Spectrin (SPEC) staining (red) visualizes the sarcolemma. DAPI staining visualizes nuclei. Scale bar 50 μm.

The definition of minimal‐invasive biomarkers of pathophysiological impact is important for patient stratification and monitoring of disease progression as well as of therapeutic intervention. To verify the impact of neurological relevant proteins identified as being dysregulated in *VWA1*‐mutant white blood cells by proteomic profiling, immunostaining studies of BET1, HNRNPDL, NEFM and PHGDH as paradigmatic proteins were carried out on cross‐sectioned quadriceps muscle biopsies derived from two unrelated *VWA1*‐patients (patient 3 and 7 from Table 1). Immunofluorescence studies of BET1 revealed a sarcoplasmic increase accompanied by the presence of focal immunoreactive dots in muscle cells of both *VWA1*‐patients in addition to a profound immunoreactivity in neurogenic targets (white arrow) (Figure 2A). Co‐immunofluorescence studies of BET1 and p62 revealed sarcoplasmic increase of both proteins with focal accumulations in terms of dot‐like immunoreactive structures of p62 and a rather general increase of BET1 in single muscle fibres, but no co‐localisation which would have been indicative for an involvement of BET1‐positive vesicles in aggregate formation (Figure S1C). Immunostaining of HNRNPDL revealed a sarcoplasmic increase accompanied by the presence of dot‐like immunoreactive structures in patient‐derived muscle cells (Figure 2B). A comparable finding was obtained for PHGDH (Figure 2C). Immunofluorescence studies of NEFM revealed a sarcoplasmic increase only in few myofibres but a considerable increased abundance in extra muscular cells localized within the extracellular matrix in muscle biopsy specimens derived from the two patients compared to controls (Figure 2D).

Given that muscular increase of PHGDH has been described in the context of both regeneration^19^ and initiation of apoptosis via the caspase‐3 pathway,^20^ additional co‐immunofluorescence studies were carried out utilising antibodies targeting neonatal myosin (muscle regeneration marker) and caspase‐3 (apoptosis marker). These studies revealed a co‐localisation of PHGDH dot‐like sarcoplasmic structures (often enriched at the sarcolemma) with caspase‐3. Enrichment of PHGDH in muscle cells immunoreactive for neonatal myosin was not observed (Figure S1C).

To investigate if BET1, HNRNPDL, NEFM and PHGDH also show altered level and distribution in other neurogenic conditions, muscle biopsy specimens derived from one genetically confirmed *BICD2*‐patient (heterozygous: c.320C>T; p.(Ser107Leu)) and two patients with idiopathic neurogenic muscular atrophy were analysed by immunostaining.

Immunofluorescence studies of BET1 revealed a sarcoplasmic increase accompanied by the presence of focal immunoreactive dots in muscle cells of both, the NMA‐patients and the *BICD2*‐patient (Figure S2). Co‐immunofluorescence studies of BET1 and p62 revealed a pronounced immunoreactivity of p62 in the neurogenic targets of skeletal muscle fibres derived from patients with idiopathic NMA. Some of these targets showed a weak co‐localisation with BET1 (white arrows) (Figure S2). Thus, in comparison to the staining results obtained in the *VWA1*‐patients, sarcoplasmic increase and BET1‐immunoreactivity of the neurogenic targets is less pronounced in the NMA patients and the *BICD2*‐case, whereas these disease controls a stronger p62‐immunoreactivity of these neurogenic targets was observed. Results of the co‐immunofluorescence studies of caspase‐3 and PHGDH confirm a dot‐like co‐immunoreactivity of these two proteins and thus are in line with the results obtained on the *VWA1*‐patients. Further co‐immunofluorescence studies of PHGDH and neonatal myosin excluded confirm the finding obtained in *VWA1*‐patients by showing no co‐localisation (Figure S2) and thus make an involvement of PHGDH in muscle cell regeneration in deneravated muscle of neuromyopathies (*VWA1*‐ or *BICD2*‐related) rather unlikely. Immunofluorescence studies of HNRNPDL in the disease control group revealed a strong immunoreactivity of neurogenic targets (white arrows) and only a generalized sarcoplasmic increase in few fibres (Figure S2) and thus are in contrast with the findings obtained in the two VWA1‐patients where a more pronounced generalized sarcoplasmic increase was identified (Figure 2). Immunofluorescence studies of NEFM in muscle biopsy specimens derived from NMA‐patients as well as from the *BICD2*‐patient revealed no sarcoplasmic increase but a considerable increased abundance in extra muscular cells localized within the extracellular matrix and thus recapitulate the findings obtained in the two *VWA1*‐patients (Figure S2).

**FIGURE 3:**
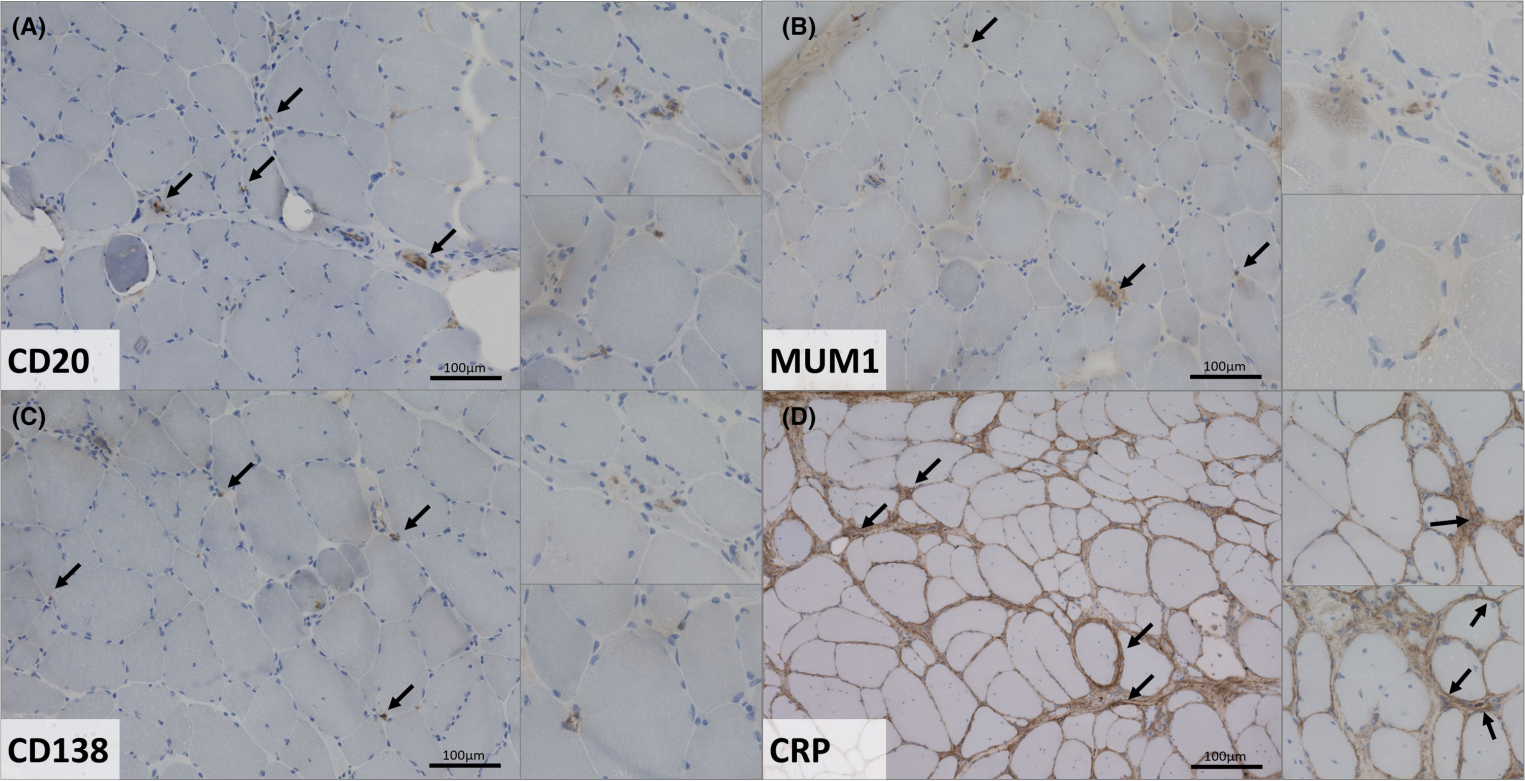
Immunohistochemistry findings in a *VWA1*‐patient derived muscle biopsy. (A) Immunostaining of CD20 revealed cells in capillaries and vessels within the muscle tissue in addition to reactivity in few circulating cells. (B) Increased immunoreactivity of MUM1^+^ cells in muscular capillaries and vessels in addition to immunoreactivity in few circulating cells. (C) CD138 immunostaining showed a reactivity of singular cells in muscular capillaries and vessels as well as in few circulating cells. (D) CRP staining revealed a pronounced immunoreactivity in the thickened connective tissue in addition to immunoreactivity in big, foamy, rounded cells (most likely macrophages).

Prompted by our combined proteomic findings showing a dysregulation of immune‐related proteins in blood (white blood cells and plasma) of *VWA1*‐patients with altered immunoglobulins in plasma indicative for altered B cell activation and inflammation (see Table S1 and Table 2), we next analysed the presence of the B cell markers CD20 and MUM1, the plasma cell marker CD138 and the inflammation marker C‐reactive protein (CRP) in the muscle biopsy specimen derived from *VWA1*‐patient 3. Immunohistochemistry studies revealed immunoreactivity of CD20^+^ and MUM1^+^ B cells in capillaries and vessels within the muscle tissue. Only few circulating cells immunoreactive for these B cell markers within the muscle tissue were identified (Figure 3A,B). The same finding was obtained for CD138^+^ plasma cells (Figure 3C). CRP immunostaining revealed a pronounced reactivity in the thickened connective tissue in addition to a reactivity in big, foamy, rounded cells (most likely macrophages) (Figure 3D).

## 4 DISCUSSION

Clinical proteomic investigations are applied to gain a better understanding of pathophysiological processes underlying in rare diseases such as neuromuscular disorders. These investigations have proven to provide an essential understanding of the cellular malfunctions and protein dysregulations contributing to phenotypical manifestation. The identification of biomarkers monitorable in a minimal‐invasive manner is an important pre‐requisite for the read‐out of therapeutic success of (new) intervention concepts. Ideally, those biomarkers are of pathophysiological relevance and are even analysable in (patient‐derived) in vitro systems to enable the testing of new interventional concepts preclinically. Hereby, biomarkers which are dysregulated in different patient cohorts with clinical overlaps may be suitable to monitor the therapeutic success of so‐called ‘basket trials’.

### 4.1 Cellular blood biomarkers of pathophysiological relevance

To systematically address the need of still lacking biomarkers of pathophysiological relevance for *VWA1*‐related neuromyopathy, a relatively novel neuromuscular condition, proteomic profiling on white blood cells was carried out. This approach led to the identification of 100 dysregulated proteins including such of neurological relevance including BET1, HNRNPDL, NEFM and PHGDH. BET1 is involved in the vesicular transport from the ER to the Golgi complex and bi‐allelic variants establish impaired vesicular transport leading to muscular dystrophy complicated by epilepsy.^21^ Confirmational immunostaining studies on muscle biopsy specimens derived from *VWA1*‐patients were carried out to examine the impact of the protein dysregulations identified in white blood cells also for skeletal muscle as a tissue clinically affected by the loss of functional WARP. The study of BET1 revealed a sarcoplasmic increase with occasional focal accumulations as well as profound immunoreactivity within the neurogenic targets in *VWA1*‐mutant muscle fibres. Of note, these immunoreactive areas do not show an overlap with areas immunoreactive for p62, a known protein aggregation marker. This microscopic finding suggests that BET1 increase is not based on the build‐up of protein aggregates and rather reflects increase of BET1‐containing vesicles in the pathophysiology of *VWA1*‐related neuromyopathy. This assumption is supported by the fact that protein aggregates in terms of the build‐up of vacuoles and myelin‐like structures was not described in muscle biopsy specimens analysed by electron microscopy.^5^ Co‐immunofluorescence studies of p62 and BET1 on muscle biopsy specimens derived from two idiopathic NMA‐patients in addition to one *BICD2*‐patient suffering from neuromyopathy revealed a less pronounced immunoreactivity of BET1 within the neurogenic targets whereas more p62‐immunoreactivity was pronounced within these structures in comparison with the two *VWA1*‐patients studied here. This finding suggests that BET1 shows a varying involvement in target formation and maturation across different muscular conditions defined by denervation. However, further studies—ideally on cohorts with defined genetic defects such as Charcot–Marie–Tooth neuropathies and hereditary forms of amyotrophic lateral sclerosis—are needed to elucidate the varying impact of BET1 in the formation of neurogenic targets more precisely.

Our microscopic studies of PHGDH confirmed an increase also in *VWA1*‐mutant muscle cells and moreover displayed a co‐localisation with caspase‐3 indicating that PHGDH‐increase is related to apoptosis. Immunofluorescence findings obtained on our disease control group support this assumption. Of note, in preclinical studies Gao and colleagues already demonstrated that activation of the PHGDH/Bcl‐2/caspase‐3 pathway promotes muscle wasting and that CCF642, a potent protein disulfide isomerases inhibitor, hereby may serve as a promising therapeutic.^20^ Given that protein folding in the endoplasmic reticulum (ER) is an oxidative process that relies on protein disulfide isomerases^22^ it is important to note that PHGDH increase was already identified in a white blood cell model of Marinesco‐Sjögren syndrome (MIM: 248800),^23^ a multisystemic disorder characterized by ER‐stress burden, myopathy and vulnerability of the peripheral nerves^24^ and that prolonged ER‐stress (which can be caused by oxidative stress) notoriously may result in initiation of apoptosis. Oxidative stress in white blood cells derived from our *VWA1*‐patients is indicated by increase of SELENOT, TXNIP, GSTM3 and ENSA (see Table S1) and affection of the ER is suggested by increase of ALG2, SSR1 as well as of VMA21 (see Table S1). The latter is an ER‐resident protein required for the assembly of the V0 complex of the vacuolar ATPase (V‐ATPase) and related to a myopathy with excessive autophagy (XMEA; MIM: 310440). Further studies on oxidative and ER‐stress are needed to decipher their exact molecular contribution to *VWA1*‐related pathophysiology.

Proteomic profiling of *VWA1*‐mutant white blood cells moreover revealed increase of HNRNPDL which is known to promote transcription activation in differentiated myotubes. Prompted by the fact that dominant *HNRNPDL* variants cause an autosomal dominant degenerative myopathy characterized by slowly progressive wasting and weakness of the proximal muscles of arms and legs accompanied by dystrophic features on muscle biopsy (LGMD D3; MIM: 609115), HNRNPDL level and distribution was also studied in muscle biopsies of *VWA1*‐patients and a disease control group (two idiopathic NMA‐cases in addition to one *BICD2*‐related neuromyopathy patient): microscopic investigation revealed sarcoplasmic increases accompanied by the presence of dot‐like immunoreactive structures in the two *VWA1*‐patients. Of note, a generalized sarcoplasmic increase of HNRNPDL was only rarely identified in single muscle fibres within our disease control group, whereas here a more pronounced HNRNPDL‐immunoreactivity within neurogenic targets was detected. This finding (also in line with the immunostaining finding obtained for BET1; see above) not supports the assumption a varying protein composition of these targets across different neurogenic and neuromyogenic conditions but moreover hint towards a more pathophysiological role of HNRNPDL in *VWA1*‐related neuromyopathy compared to the other disease conditions studied here. Thus, one might postulate that HNRNPDL holds the potential to serve as a characteristic biomarker of pathophysiological relevance in *VWA1*‐related neuromuscular phenotypes. However, studies on white blood cells and muscle biopsy specimens derived from larger patient cohorts a crucial to draw final conclusions and to claim specificity. Albeit, it has to be taken into consideration that rare diseases are especially challenging to establish biomarkers based on the frequency of the respective diseases in combination to the availability of biomaterial needed for robust biomarker research. This in fact is certainly a general limitation for the neuromuscular field especially regarding nano‐rare diseases such as *VWA1*‐related neuromyopathy. Nevertheless, regarding the pathophysiological impact of HNRNDPDL it is important to note that acceleration of intrinsic HNRNPDL self‐aggregation (caused by mutations of the prior‐like domain) was already described and discussed to presumably contribute to the pathogenesis of LGMD D3.^25^ However, further functional studies such as immunoprecipitation of HNRNPDL and subsequent proteomics would be needed to further elucidate the pathophysiological relevance of the observed pathological distribution of HNRNPDL in *VWA1*‐related neuromyopathy.

Neurofilaments (NFs) are the most abundant cytoskeletal component of vertebrate myelinated axons determining axonal calibre, promoting axonal growth and forming a 3‐dimensional lattice towards organisation of cytoplasmic organelles. Of note, stoichiometry of NF protein subunits (NEFL, NEFM and NEFH) has to be tightly controlled to avoid the formation of NF neuronal cytoplasmic inclusions, axonal degeneration and neuronal death, all pathological hallmarks of motoneuron disorders such as amyotrophic lateral sclerosis (ALS).^26^ NEFM (Neurofilament Medium) polypeptide is increased in white blood cells derived from *VWA1*‐patients. However, immunofluorescence studies on muscle biopsy specimens derived from *VWA1*‐patients and disease controls did not reveal an assumed increase and aggregation in mutant muscle cells but showed an increase in extra‐muscular cells. Although muscle cell vulnerability has already been directly linked to pathological variants affecting NEFL (as another NF subunit),^27^, ^28^ this finding more likely indicates that NEFM may be involved in a generalized myopathological tissue remodelling driven by non‐muscle cells upon denervation of the presence of a neuromyogenic condition rather than by increase in myofibres itself. Further co‐immunostaining studies are crucial to decipher the nature of extra‐muscular cells presenting with increased NEFM abundance in (*VWA1*‐ and *BICD2*‐related) neuromyopathies and neurogenic muscular atrophies. However, one might speculate that the increased NEFM abundance in white blood cells is perhaps more indicative for pathophysiological processes taking place in the nervous system rather than in the musculature of *VWA1*‐based neuromyopathy. Future studies on nervous tissue are needed to proof this assumption but might be challenging regarding the availability of this biomaterial derived from *VWA1*‐patients (also by taking the rarity of this disease into consideration).

### 4.2 Plasma biomarkers of pathophysiological relevance

To identify minimal invasive biomarkers for *VWA1*‐related neuromyopathy beyond such investigable in cellular parts of blood like white blood cells, we moreover analysed the proteomic signature of plasma samples derived from same patients. This approach led to the identification of dysregulated 15 proteins (11 increased and 4 decreased). Interestingly, four of the increased proteins (CA1, CA2, CAT and PRDX2) display antioxidative function. Of note, CA1 has already been described as serum biomarker in dystrophin deficient muscular dystrophy.^29^ CA3, another carbonic anhydrase, known as a recurrent serum marker in dystrophin deficient muscular dystrophy,^30^ was not significantly dysregulated in serum derived of our patients. Proteomic profiling on different mouse models identified increased CA2 in aging and neurodegeneration.^31^ Thus, one might postulate that the increased abundance of CA1 and CA2 in plasma samples derived from our *VWA1*‐patients also accords with degenerative processes affecting skeletal musculature and nervous tissue. Notably, application of carbonic anhydrase inhibitors displayed a therapeutic potential in two animal models of dystrophin deficient muscular dystrophy.^32^

TSP1 is an extracellular glycoprotein mediating cell‐to‐cell and cell‐to‐matrix interactions and was found to be increased 2.01‐fold in *VWA1*‐patient derived plasma. TSP1 has already been described to correlate with macrophage activity and disease progression in dysferlin deficient mice^33^ and to mediate muscle damage in brachio‐cervical inflammatory myopathy and systemic sclerosis.^34^ However, although TSP1 has been highlighted to serve a blood biomarker in a diversity of disorders such as peripheral arterial disease^35^ and precapillary pulmonary hypertension,^36^ to the best of our knowledge, thus far circulating TSP1 has not been described a blood biomarker (candidate) in neuromuscular diseases with *VWA1*‐related neuromyopathy being the first one. In light with the antioxidative potential of CA1, CA2, CAT and PRDX2 also the known function of TSP1 in antagonization of nitric oxide‐stimulated vascular smooth muscle cell response^37^ hints towards a more generalized increase of antioxidative blood biomarkers in *VWA1*‐related neuromyopathy. This assumption is moreover supported by the identification of increased plasma level of CRP, an acute inflammatory protein which is among others synthesized in muscle and known to be involved in nitric oxide release and apoptosis.^38^ A meta‐analysis revealed that sarcopenia (which is known to be accompanied by increased of muscle fibrosis) seems to be associated with elevated serum CRP levels.^39^ Immunostaining studies performed on sections of a muscle biopsy derived from one *VWA1*‐patient revealed a pronounced CRP‐increase in the extracellular matrix. Thus, one might assume that increased plasma level of CRP accord with myopathological processes underlying in *VWA1*‐related neuromyopathy. Given that the proteomic signature of plasma derived from *VWA1*‐patients also revealed decreased abundance of immunoglobulins suggestive for altered B and plasma cell activation (Table 2), immunostaining was carried out for CD20, MUM1 and CD138 on muscle sections. However, only few immunoreactive cells were found and studies on further biopsies are needed to evaluate the potential involvement of B cell and plasma cell activation in *VWA1*‐related muscle pathology. Along this line, further studies on a larger cohort of *VWA1*‐patients are needed to validate the potential of these circulating proteins to serve as robust blood biomarkers of *VWA1*‐related neuromyopathy. Investigation of longitudinal samples would hereby address the potential to also serve as a disease progression marker. Immunostaining studies are needed to clarify their definite role in the cellular pathophysiology of *VWA1*‐related neuromyopathy i.e. in the context of oxidative stress burden (already indicated by the proteomic signature of *VWA1*‐mutant white blood cells) and the modulation of inflammatory processes as well as in fibrotic remodelling.

Regarding these plasma biomarker candidates, it should be mentioned that plasma biomarkers might potentially be confounded by other factors such as age, renal function, BMI (body volume), physical activity, among others and a correction for these potential confounders was not carried out in our study but should be part of potential further validation studies on larger cohorts.

## 5 CONCLUSIONS

Results of our combined studies allowed the identification of protein dysregulations in blood (white blood cells and plasma) with some proteins affected harbouring known relevance for pathophysiological processes taking place in muscle cells. Further immunofluorescence studies of four of these markers, BET1, HNRNPDL, PHGDH and NEFM on muscle biopsy specimens derived from two *VWA1*‐, one *BICD2* and two NMA patients unveiled HNRNPDL as a marker showing differences between *VWA1*‐patients and patients suffering from *BICD2*‐related or idiopathic neuro(myo)genic conditions. Thus, HNRNPDL may hold the potential to serve as a minimal invasive biomarker with pathophysiological for *VWA1*‐related neuromyopathy enabling new concepts in patient stratification and the monitoring of therapeutic intervention concepts.

## AUTHOR CONTRIBUTIONS

**Mohammed Athamneh:** Resources (equal). **Nassam Daya:** Data curation (equal); methodology (equal); visualization (equal). **Andreas Hentschel:** Formal analysis (equal); methodology (equal); visualization (equal). **Andrea Gangfuss:** Investigation (equal); resources (equal). **Tobias Ruck:** Supervision (equal); writing – review and editing (equal). **Adela Della Marina:** Resources (equal). **Ulrike Schara‐Schmidt:** Conceptualization (equal). **Albert Sickmann:** Formal analysis (equal). **Anne‐Katrin Güttsches:** Resources (equal). **Marcus Deschauer:** Investigation (equal); resources (equal); writing – review and editing (equal). **Corinna Preusse:** Formal analysis (equal); investigation (equal); methodology (equal); validation (equal). **Matthias Vorgerd:** Conceptualization (equal); resources (equal); writing – review and editing (equal). **Andreas Roos:** Conceptualization (equal); investigation (equal); supervision (equal); writing – original draft (equal).

## FUNDING INFORMATION

This study was supported by the ‘Ministerium für Kultur und Wissenschaft des Landes Nordrhein‐Westfalen’, the ‘Regierenden Bürgermeister von Berlin‐Senatskanzlei Wissenschaft und Forschung’, and the ‘Bundesministerium für Bildung und Forschung’. The European Regional Development Fund (ERDF) financed parts of this study in the framework of the NMD‐GPS project (https://nmd‐gps.net/). A.R. received funding from The French Muscular Dystrophy Association (AFM‐Téléthon; grant: 21644). The financial support of the German Society of Muscular Diseases (DGM) is also gratefully acknowledged (Libi‐NME grant to A.R., M.V. and U. S‐S.).

## CONFLICT OF INTEREST STATEMENT

The authors declare to not have any conflict of interest.

## DISCLOSURES

There are no disclosures relevant to this manuscript.

## Supporting information


Figure S1:

Figure S2:

Table S1.

## Data Availability

The mass spectrometry proteomics data have been deposited to the ProteomeXchange Consortium via the PRIDE^18^ partner repository with the dataset identifier PXD040226.
