# Supplementary material for: Proteomic studies in VWA1‐related neuromyopathy allowed new pathophysiological insights and the definition of blood biomarkers
Source: J Cell Mol Med. 2024 Apr 23;28(8):e18122. doi: 10.1111/jcmm.18122 (PMC11037410; doi:10.1111/jcmm.18122)
Supplement: Supplementary file 1 — Figure S1: [file JCMM-28-e18122-s003.pptx]

## Slide 1
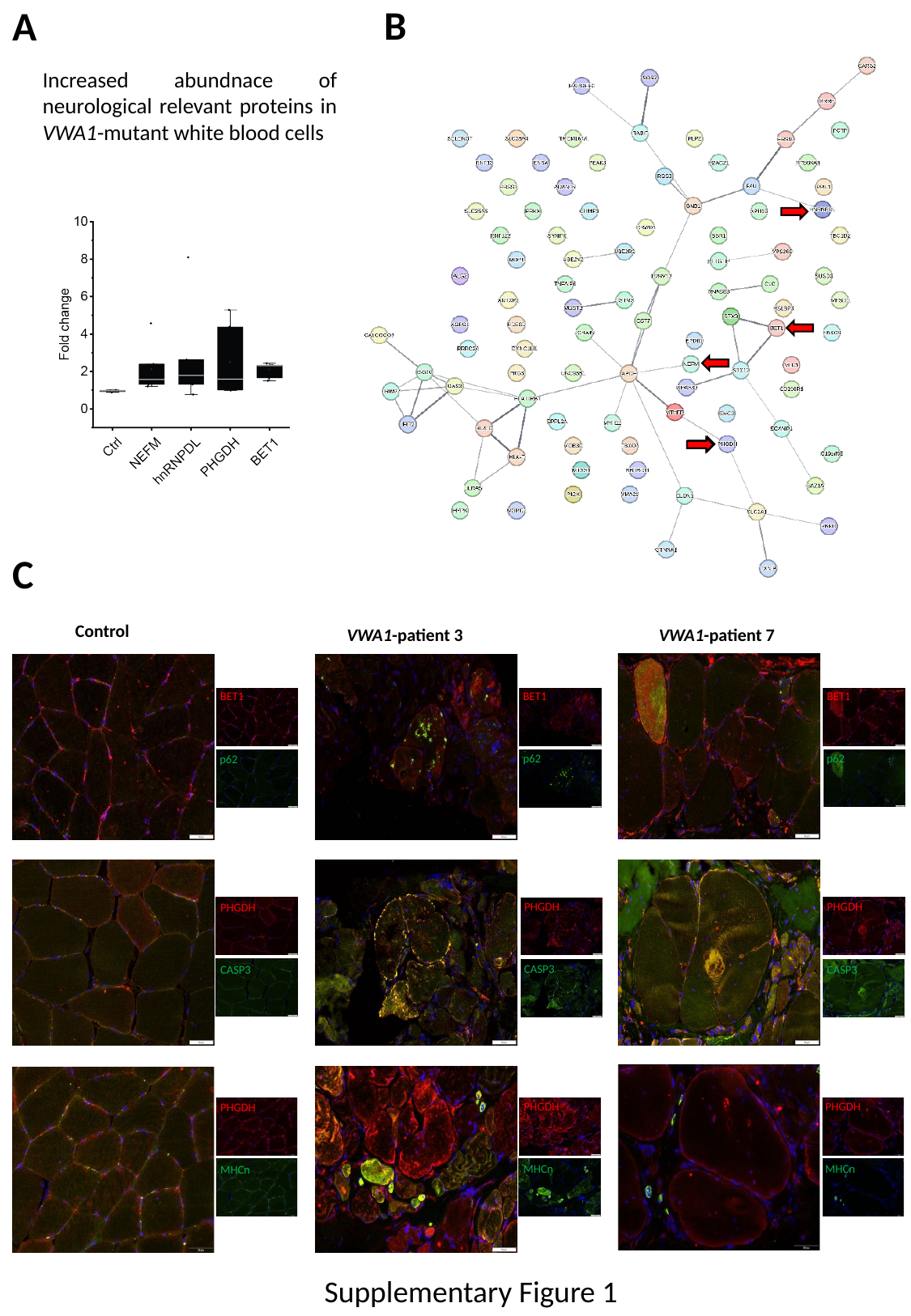

A
B
Increased abundnace of neurological relevant proteins in VWA1-mutant white blood cells
C
Control
VWA1-patient 3
VWA1-patient 7
BET1
BET1
BET1
p62
p62
p62
PHGDH
PHGDH
PHGDH
CASP3
CASP3
CASP3
PHGDH
PHGDH
PHGDH
MHCn
MHCn
MHCn
Supplementary Figure 1
