# Supplementary material for: Proteomic studies in VWA1‐related neuromyopathy allowed new pathophysiological insights and the definition of blood biomarkers
Source: J Cell Mol Med. 2024 Apr 23;28(8):e18122. doi: 10.1111/jcmm.18122 (PMC11037410; doi:10.1111/jcmm.18122)
Supplement: Supplementary file 2 — Figure S2: [file JCMM-28-e18122-s001.pptx]

## Slide 1
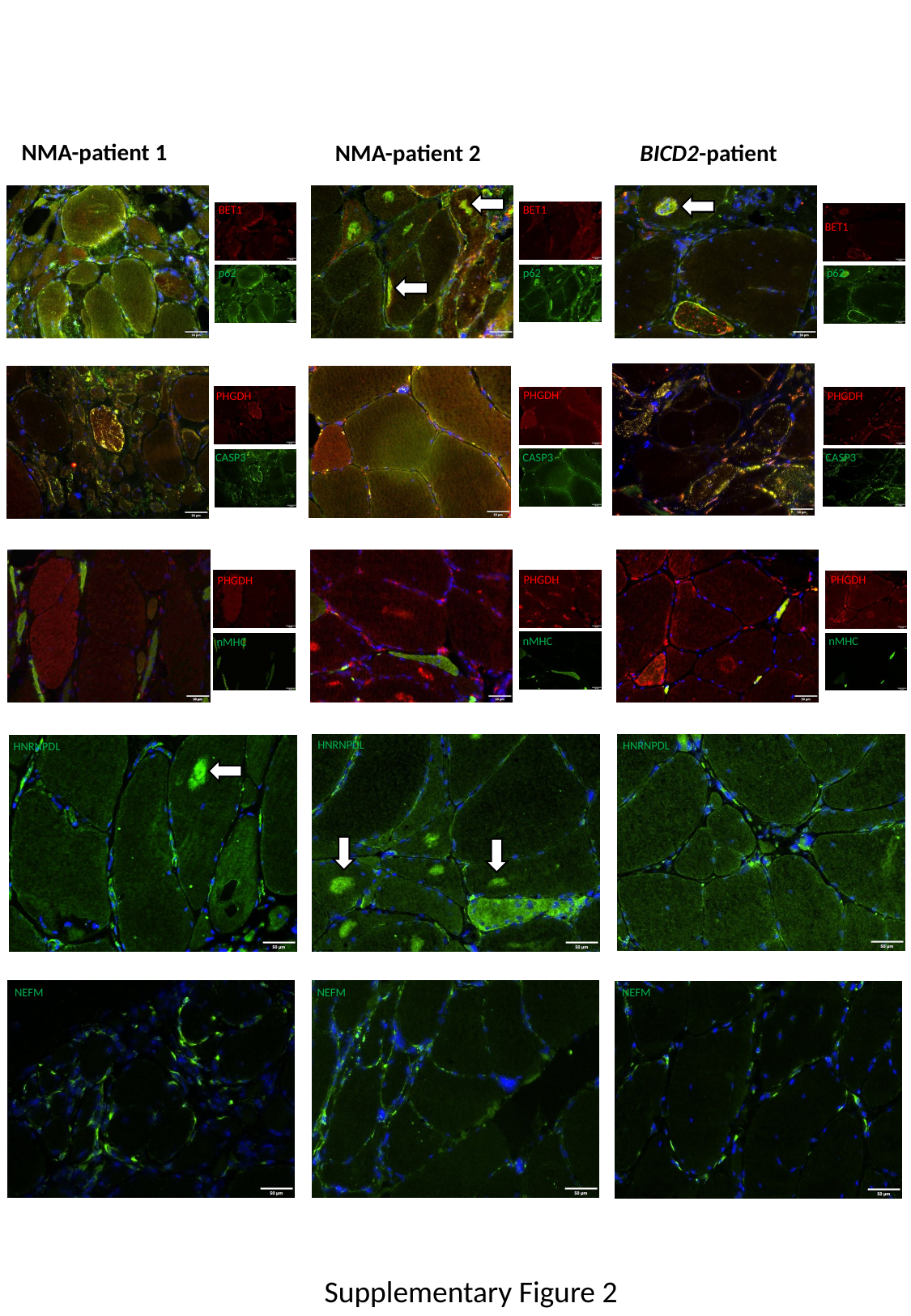

NMA-patient 1
NMA-patient 2
BICD2-patient
BET1
BET1
BET1
p62
p62
p62
PHGDH
PHGDH
PHGDH
CASP3
CASP3
CASP3
PHGDH
PHGDH
PHGDH
nMHC
nMHC
nMHC
HNRNPDL
HNRNPDL
HNRNPDL
NEFM
NEFM
NEFM
Supplementary Figure 2
